# Keying Into Cognition: Temporal Smoothing of Smartphone Typing Behaviors for Passive Assessment of Processing Speed and Executive Function in Individuals With Mood Disorders

**DOI:** 10.1007/s12559-026-10549-y

**Published:** 2026-04-01

**Authors:** Mindy K. Ross, Theja Tulabandhula, Theresa M. Nguyen, Emma Ning, Sarah Kabir, Andrea T. Cladek, Amruta Barve, Ellyn Kennelly, Faraz Hussain, Jennifer Duffecy, Scott A. Langenecker, John Zulueta, Alexander P. Demos, Olusola A. Ajilore, Alex D. Leow

**Affiliations:** 1 Department of Psychiatry, University of Illinois Chicago, Chicago, IL 60612 USA; 2 Department of Biomedical Engineering, University of Illinois Chicago, Chicago, IL 60612 USA; 3 Department of Information and Decision Sciences, University of Illinois Chicago, Chicago, IL 60612 USA; 4 Department of Psychology, University of Illinois Chicago, Chicago, IL 60612 USA; 5 Department of Psychiatry and Behavioral Health, The Ohio State University, Columbus, OH 43210 USA; 6 Department of Computer Science, University of Illinois Chicago, Chicago, IL 60612 USA

**Keywords:** Cognitive assessment, MHealth, Digital phenotyping, Smartphones

## Abstract

Cognitive deficits commonly affect everyday life for individuals with mood disorders, even between mood episodes. Monitoring of these symptoms can pose several challenges due to the limitations of current methods, prompting the need for enhanced modalities to unobtrusively and objectively measure cognitive function and its fluctuations in individuals. This study explored the feasibility of passively assessing processing speed and executive function, traditionally measured by the trail-making test part B (TMT-B), using smartphone keyboard typing behaviors and assessed how diurnal patterns may impact cognitive function. Through a novel method of temporal smoothing of smartphone typing behaviors via graph-regularized singular value decomposition, we engineered features to capture typing regularity as a proxy for diurnal patterns and sleep. These features were added to machine learning models constructed to predict TMT-B performance and evaluated for improvement in model performance. Of the models tested, a random forest model built with the addition of typing regularity features performed the best with the lowest RMSE and MAE of 0.769 and 0.644, respectively. Our findings suggest that aspects of individuals’ cognitive function, specifically processing speed and executive function, can be estimated through their smartphone typing behaviors without the need for clinical or demographic input, and these estimates are improved with additional information capturing diurnal patterns and estimated sleep. This objective approach, passively administered in-the-wild, has the potential to supplement current methods of cognitive assessment and provide a more detailed report of cognitive fluctuations and the influence of diurnal patterns on cognitive function in individuals with mood disorders.

**Supplementary Information:**

The online version contains supplementary material available at 10.1007/s12559-026-10549-y.

## Introduction

Mood disorders are highly prevalent with approximately 20% of the US population experiencing a mood disorder at some point during their lifetime [1]. Moreover, in 2021, approximately 5% of the global population were living with bipolar disorder or a depressive disorder, the predominant categories of mood disorders [2, 3]. The symptoms of these disorders center around persistent deviations in mood, either abnormally depressed and/or elevated, that cause significant impairments to everyday functioning. Depressive disorders are characterized by a period of at least two weeks of depressed mood and decreased interest in once pleasurable activities along with possible feelings of worthlessness or guilt, changes to concentration and attention, appetite, sleep, psychomotor activity, or energy, or thoughts of death or suicidal ideation [2, 4]. Bipolar disorders comprise of a cycling between depressive episodes, described above, and manic episodes, which are characterized by a period of at least 1 week of unnaturally elevated or irritable mood, increased activity or energy, and may include symptoms related to racing thoughts, increased talkativeness, self-esteem, reckless behavior, sexual drive, sociability, or goal-directed activity, or decreased need for sleep [2, 4]. While for some, these disorders consist of a single episode, relapsing symptoms commonly occur for many individuals. As such, long-term follow-up and assessments by trained mental health professionals can be necessary to effectively manage these disorders.

In addition to disturbances in mood, cognitive complaints are also common, specifically in areas of memory, attention, and executive function [5, 6]. Though there is some dispute as to the precise prevalence and severity of cognitive symptoms in this population [7, 8], cognitive deficits have been reported to affect everyday functioning and may even persist during periods of euthymia [5, 6]. Often, this cognitive dysfunction has been found to worsen with increased mood symptom severity [5]. Cognitive deficits are generally assessed periodically using a battery of neuropsychological tests. However, these tests are just snapshot assessments conducted outside of naturalistic settings, with some being further subject to practice effects [9–11]. While self-reports are taken into consideration in attempt to fill in the gaps between assessments, these reports are impacted by recall biases and may not convey an accurate portrayal of the impairments [12].

As such, further supplementing the standard of care could greatly help cognitive symptom monitoring for these disorders. To attempt to remedy the issues seen with traditional assessments, ecological momentary assessments (EMAs) have become popular in recent years due to the more granular data recorded with minimal impact by recall bias [13, 14]. EMAs consist of surveys and assessments (i.e. mood questionnaires or neuropsychological tests) that are completed, sometimes digitally, on a regular basis in the individual’s natural environment [14]. However, these real-time assessments still require active input from the individual, which can become time consuming, is reliant on compliance, and is still affected by response and recall biases [13, 14].

To address these issues, an unobtrusive in-situ approach to objective assessments of cognitive symptoms would be an ideal method to elucidate the everyday impairments experienced by those with mood disorders without increasing the burden on individuals. With the rise in smartphone usage across the population, these devices have begun to be integrated into the development of novel assessment methods [15–17]. Analyses of phone usage patterns and the data collected from integrated sensors have shown promise to monitor the symptoms of mood disorders [18–22], although many studies rely on continuous recording and tracking of information such as GPS location that has led to concerns with diminished phone battery life and privacy [23]. Partially to address these concerns, other studies have focused on analyzing patterns derived solely from smartphone typing dynamics to assess symptomatology [24–29]. Through detecting changes in participants’ usual typing behavior, information about their underlying cognitive state might be revealed [30], with previous work suggesting that typing speed and other keyboard dynamics may serve as a proxy for processing speed [30–32]. The information extracted in-the-wild might also serve as a better reflection of the functional cognitive state of the individual and identify more granular fluctuations in cognitive performance than traditional assessment methods. Furthermore, the longitudinal nature of the data collection could contribute towards more personalized and predictive approaches, which would greatly help symptom management through early detection of possible changes [33–35].

Unlike most studies, here we further take into consideration the effect of individual diurnal patterns on cognitive performance, since disruptions in circadian rhythm and sleep are a well documented component of mood disorders with disturbances in sleep listed as one of the possible criteria for the diagnosis of a mood disorder [4, 36, 37]. Indeed, individuals with depression commonly report sleep symptoms relating to either insomnia or hypersomnia [38]. As with cognitive symptoms, these symptoms have also been found to worsen with increased mood symptoms and do not resolve entirely during an euthymic state [39, 40]. It has been hypothesized that increases in sleep disturbances may indicate an early warning sign for the precipitation of a mood episode, but this may be dependent on many factors including the type of forthcoming episode as well as which mood disorder the person has [41, 42]. Additionally, several theories exist regarding the causality between sleep disturbances and mood episodes, but the exact mechanisms surrounding the two have yet to be resolved [37]. Cognitive impairment and sleep disturbances are both experienced by those with mood disorders, but the relationship between the two is not well understood. Pearson et al. summarized some previous studies that suggested there are significant associations between self-reported sleep disturbances and cognitive impairment in preliminary findings [43]. More precisely, a study by Cabanel and colleagues found that poor self-reported sleep quality negatively affected performance on the TMT-B in those with major depressive disorder compared to health controls [44].

Information regarding diurnal rhythms and sleep are typically obtained through self-reports or objective measures such as actigraphy or polysomnography. These methods vary in accuracy with self-reports being prone to bias and objective measures requiring more active, and sometimes extensive, participation [43]. Of the objective measures, polysomnography is considered to be the gold standard for measuring sleep quantity and quality, but its high cost and intrusiveness limit its practicality for continuous data collection in naturalistic settings [43, 45]. As a result, many researchers have turned to wearable devices (e.g. smartwatches) for continuous daily activity and sleep monitoring using sensors such as the accelerometer [46]. While wearable devices offer a passive modality to monitor sleep and daily activity levels in-the-wild, this method still requires active compliance that may be beyond daily routine to continuously wear and maintain battery levels on the wearable device. To circumvent these issues, some researchers have investigated the use of phone activity, either through keyboard typing, touchscreen interactions, or screen on/off times, to extract sleep duration estimates [47–49]. Druijff-van de Woestijne and colleagues observed overall agreement between self-reported resting periods and the last and first keyboard entries surrounding a nightly absence of keystrokes [49]. Their study, however, was performed in a student population for only one week. Furthermore, daily activity patterns were not evaluated.

To this end, we developed a method to frequently assess aspects of cognition and the influence of diurnal patterns on performance using passively-collected data from smartphone keyboard typing. We chose to focus on the trail making test part B (TMT-B), which has shown worse performance in those with mood disorders compared to healthy controls [50, 51]. The TMT-B is a well-validated cognitive assessment commonly used by clinicians to measure visual attention, processing speed, and set-switching as aspects of executive functioning [10, 52], but suffers from practice effects after repeated administrations with these effects seen up to a year following the initial administration [9, 53, 54]. As such, inclusion of the TMT-B in EMAs would not be a viable approach to frequently measure these aspects of cognition despite reliability between the traditional paper version and digital adaptations [31, 55, 56]. Instead, a novel method to reliably assess these aspects of cognition would be needed to more closely monitor fluctuations in cognitive symptoms. By employing digital modalities, assessments of cognitive performance outside of a controlled environment could allow us to examine how the functional impairments may be experienced in everyday life as opposed to quantifying the exact nature of the cognitive domain without consideration for ecological validity [33, 57]. We reason that cognitive functions required to complete the TMT-B (i.e. visual attention, processing speed, and set-switching) also apply in smartphone typing during sentence formation, which can incorporate complex combinations between alphanumeric, backspace, punctuation, and special character keystrokes across multiple keyboard pages [58].

This study enhances an analysis which observed significant associations between typing speed, depression severity, and digital TMT-B (dTMT-B) performance in a small sample of individuals with and without bipolar disorder [31]. First, we present a method using smartphone typing activity to capture estimates of diurnal patterns and sleep, using surrounding patterns to supplement the inherent sporadic activity. Through models developed to predict processing speed and executive functioning otherwise measured by the TMT-B, we observed improved model performance following the addition of features engineered from these diurnal pattern approximations.

## Methods

### Participants

Participants were recruited from the greater Chicago area as part of a larger NIH study investigating the feasibility of passively monitoring mood and cognition (ClinicalTrials.gov Identifier: NCT04358900), described in detail by Ning and colleagues [32]. Briefly, individuals 25 to 50 years of age with and without a mood disorder and who owned a smartphone were eligible for the study if they did not have any of the following: active suicide ideation or a suicide attempt within the last 3 months, severe cognitive impairment, alcohol and/or substance use disorder, contraindications to MRI, any major medical or neurologic condition that would interfere with study adherence or findings interpretability, and were not pregnant, trying to become pregnant, or lactating. Informed consent was obtained from all participants who met the eligibility criteria prior to enrollment. Mood disorder diagnoses were verified by a trained clinician using the Mini International Neuropsychiatric Interview (MINI) to confirm one of the following DSM-5 disorders: bipolar disorder type I or II, major depressive disorder, persistent depressive disorder, or cyclothymia [4]. All procedures performed were first reviewed and approved by the University of Illinois Chicago Institutional Review Board (reference number 2019 − 1333, 2020/02/09).

### Data Collection

**Fig. 1:**
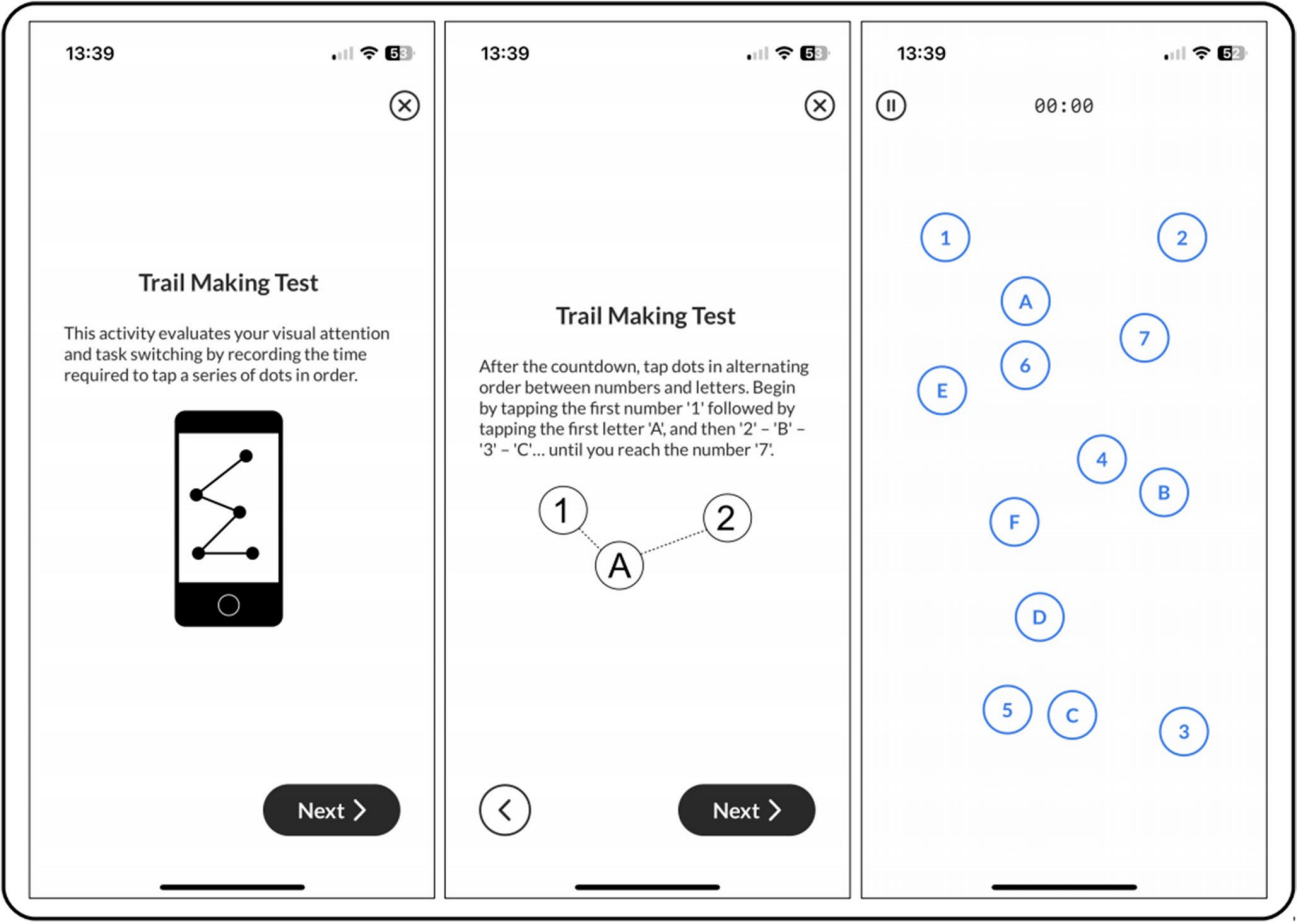
Screenshots of the dTMT-B on the BiAffect app showing the welcome screen, directions, and one example task

Participants with iPhones were instructed to download the BiAffect app and complete the dTMT-B (Fig. 1) through the app once per week for the duration of the study. In the task, individuals were instructed to connect circles containing letters and numbers in alternating and ascending order (i.e. 1-A-2-B-3-C...) by tapping their finger through the sequence as quickly as possible over a total of 13 circles. If any errors were made, the incorrectly tapped circle would turn red, and a correction by tapping the correct circle would need to be made before the participant could continue in the sequence. The timestamp, total time to completion, and number of errors were recorded. There were 12 variations of the layout of the circles on the phone screen to prevent memorization of the sequence on the phone screen. In addition to the dTMT-B, participants were also asked to complete the Patient Health Questionnaire (PHQ) without the suicidality question through the BiAffect app, which is a well-validated self-report of depression severity [59]. The PHQ presents 8 statements (plus a statement regarding suicidality not included in this study) for which the participants were asked to report to what degree they were bothered within the last two weeks by each of the statements. The suicidality item was not included in this study due to ethical concerns since the data collected was not actively monitored. Lastly, typing and accelerometer metadata (including timestamps and keypress category but not the actual text) was collected passively whenever participants typed on their iPhones using the BiAffect custom keyboard. Accelerometer readings were collected in the x-, y-, and z-axes by the phone’s embedded tri-axial accelerometer to record the phone’s acceleration and impact of gravity on the three specific axes.

### Diurnal Pattern Estimation

#### Typing Activity Matrices

An inherent problem with using smartphone typing data to infer diurnal patterns and sleep wake cycle is missingness. To illustrate this point, imagine that Alice on a specific day noticed that Bob was not using his phone during 19:00–21:00. Alice, however, should still infer that Bob was unlikely already in bed if she had one of the two following pieces of information: (1) that Bob used his phone shortly before 19:00 and shortly after 21:00 on that day, or (2) that Bob uses his phone during the same 19:00–21:00 window on all other days. That is to say, diurnal patterns should be “smoothed” not only within each day but also across different days.

**Fig. 2:**
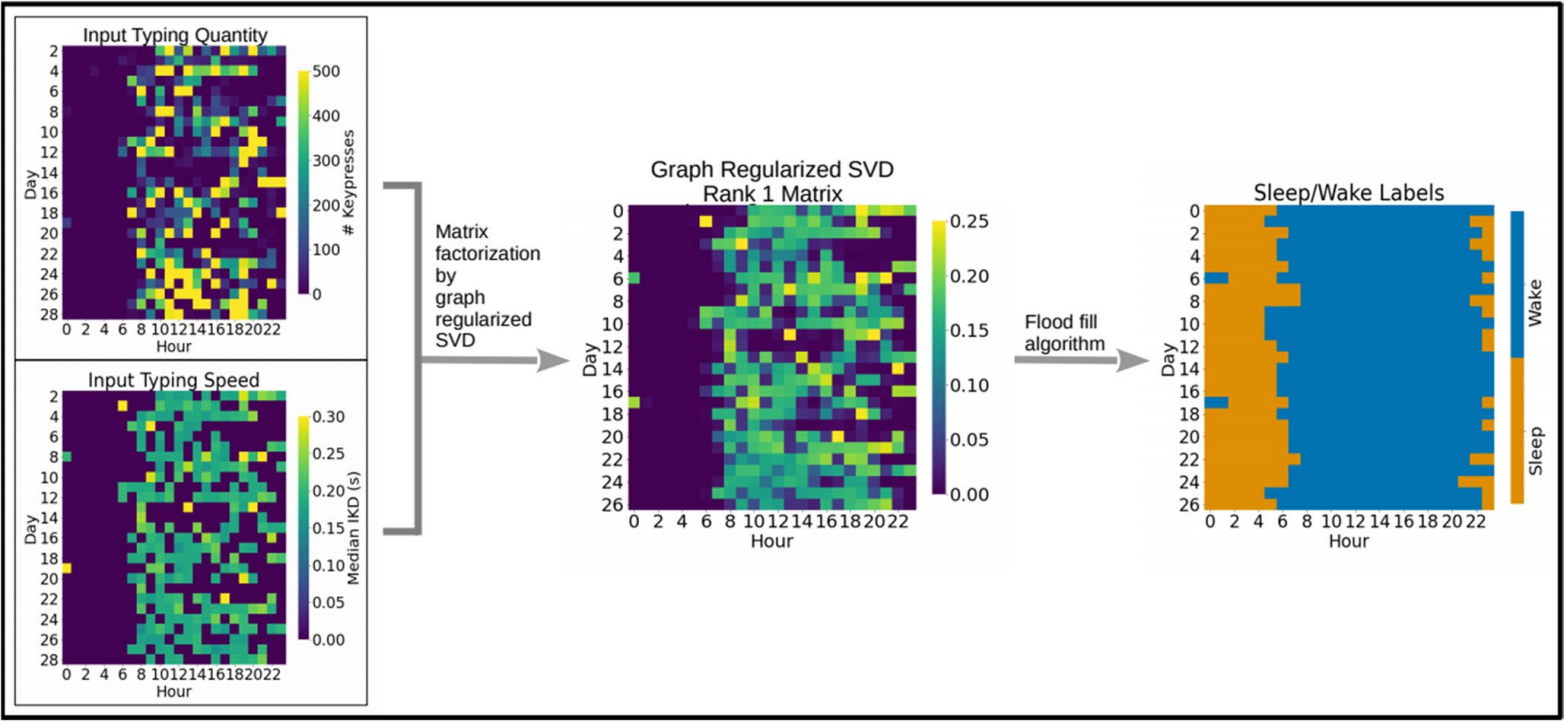
Diagram outlining the steps to obtain typing regularity and estimated sleep patterns using graph regularized singular value decomposition (SVD) of smartphone typing activity

**Fig. 3:**
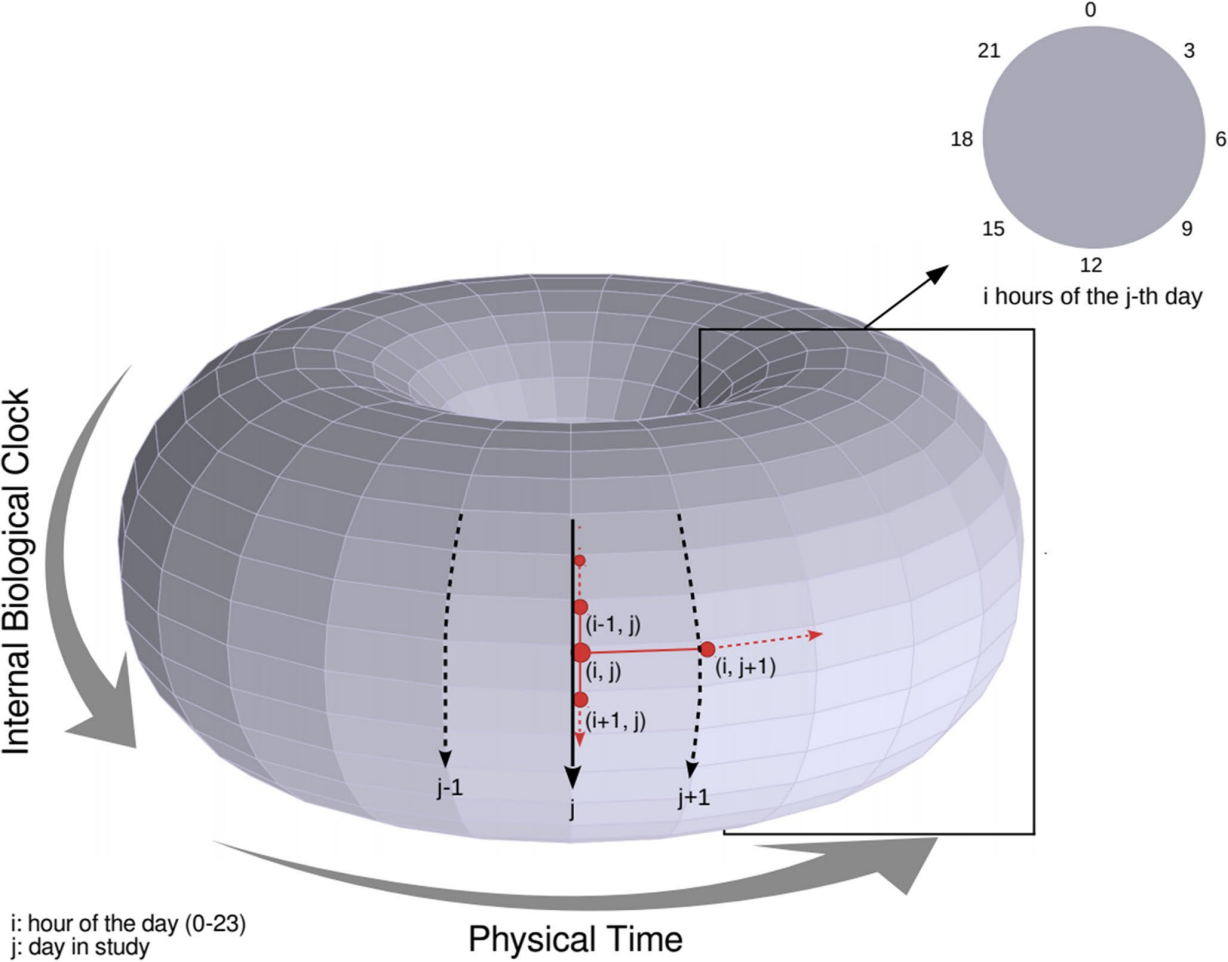
Diagram illustrating how diurnal patterns can be modeled by the surface of a torus

Given this intuition, a method was developed to capture individual diurnal patterns and estimated sleep through typing activity alone, outlined in Fig. 2. First, under the assumption that a person’s internal biological clock (subserving the observable diurnal activities) is stable, we note that the internal biological clock thus can be represented as a phase angle of a circle, that is further rotating around a large circle representing the physical clock (exactly every 24 h). In other words, mathematically speaking, the biological clock (small cycle) is “coupled” to the physical clock (large cycle) and thus can be conceptualized as a ‘flow’ or trajectory on the surface of a torus (in the case when the biological clock is exactly coupled to the physical clock, then the trajectory is periodic and returns to the same starting point every 24 h), illustrated in Fig. 3. This conceptualization thus allows us to define a toroidal temporal smoothing algorithm by utilizing a graph regularized SVD (where the graph captures the underlying toroidal representation) to infer the sleep-wake cycle based on keyboard data.

In this analysis, we describe estimated or predicted sleep as a prolonged continuous set of hours without smartphone typing activity typically during nighttime hours. These values were entirely predictive as we did not have ground truth data regarding participants’ activities during their predicted “sleep” hours. Various measures of hourly typing activity were obtained for each individual. Here, we used *Number of Keystrokes* and *Typing Speed*, but our method could accommodate any number of typing related features. Both of these measures of typing activity were arranged into matrices with the dimension of *days* × *hours*, and missing cells were filled with zero such that each matrix had a total of 24 columns for each hour of the day. To remove possible incomplete typing records during the first and last days of the study, the corresponding rows of each matrix were removed. Filtering was also performed to remove participants or weeks within participant data without sufficient typing data, since individuals with sparse typing activity resulted in poor estimations of diurnal patterns and sleep. Determined by an empirical approach to identify the minimum amount of data for reasonable predictions, participants with less than an average of 20% of the hours per day containing typing activity or less than a median amount of 50 keystrokes typed per day were excluded from analysis. These thresholds were also applied to 7 day groupings within each participants’ month of contributed data. Groupings with less typing activity than specified above, as determined by a sliding window of 7 days with one day gaps between windows, were removed from typing activity matrices prior to analysis of typing regularity. Participants with less than 7 days total following all filtering were also excluded from analysis. Lastly, the typing quantity matrix was normalized by the total number of keystrokes per individual.

#### Graph Regularized Singular Value Decomposition

Using the intuition described above in the Alice and Bob example as well as in Fig. 3, the hourly typing features Number of Keypresses and Typing Speed were combined and decomposed using graph regularized singular value decomposition (graph regularized SVD) as described by Vidar and Alvindia [60] and modified by Donelli [61] to analyze the patterns in typing activity while allowing influences from neighboring hourly and daily typing activity. Temporal smoothing via graph regularization of the SVD was performed using a weighted adjacency matrix composed of all neighboring hours and days, taking into account the circular nature of time between the hours of 23:00 and 00:00. The edge weights were calculated on the matrix containing typing quantities using Eqs. 1 and 2, respectively, where k_*i, j*_ and k_*i+1,j*_ are the keystroke quantities for neighboring hours of the j-th day and k_*i, j*_and k_*i, j+1*_ are the keystroke quantities in the i-th hour of the current j-th and next neighboring days, chronologically. The hour-edge exists with hour weight[_(i, j),(i’,j)]_ between observation (i, j) and (i’,j) if |i – i’| = 1, and the across day-edge exists with day weight[_(i, j),(i, j’)]_ between observation (i, j) and (i, j’) if |j-j’| ≤ 3. Equation 1 shows the average typing activity between neighboring hours, while Eq. 2 shows smoothing within the same hour across days in a one week window, under the assumption that biological clock is stable within that time window. The following choices were used for the hour and day edge weights:

1 $$hourweight_{\left[\left(i,\;j\right),\;\left(i',\;j\right)\right]}=\frac{k_{i,\;j}+k_{i+l,\;j}}2$$

Boundary condition: $$hourweight_{\left[\left(23,\;j\right),\;\left(0,\;j+1\right)\right]}$$$$=\frac{k_{23,\;j}+k_{0,\;j+1}}2$$

2 $$\begin{array}{l}dayweight_{\left[\left(i,\;j\right),\;\left(i',\;j\right)\right]}=median\\\left(k_{i,\;j-3},\;k_{i,\;j-2},\;k_{i,\;j-1},\;k_{i,\;j},\;k_{i,\;j+1},\;k_{i,\;j+2},\;k_{i,\;j+3}\right)\\\left\{i\in\mathbb{N}\lor0\leqslant i\leqslant23\right\}\\\left\{j\in\mathbb{N}\lor1\leqslant j\leqslant total number of days with available data\right\}\end{array}$$

To obtain the day weight, we opted to find the median keypress quantity across many neighboring days, as opposed to the mean quantity across two neighboring days, in order to extract the typical typing activity at that hour with minimal influence by isolated, abnormal typing events.

Proper matrix multiplication required that the two typing activity matrices were reshaped to be of size *observations* × *1* with the number of observations equal to the total number of days and hours with typing activity. These matrices were then stacked to form one matrix of size *observations* × *2* as the data matrix *X* for graph regularized SVD.

Graph regularized SVD was computed on the data matrix *X* and associated weighted adjacency matrix *B* using the formula described by Vidar and Alvindia [60] (reproduced below in Eq. 3) such that *X ← HW*, *α* was the scaling factor, and *r* was the rank for the factorization.

3 $$\begin{array}{l}min_{H,\;W}\left\|X-HW\right\|_F^2+\alpha Tr\left(WBW^t\right)\\subject\;to\;H^TH=I_r\end{array}$$

One modification to the derivation was used to obtain the final formulas as described by Donelli and reproduced in Eqs. 4 and 5 [61], where$\:\stackrel{\sim }{E},\stackrel{\sim }{\varSigma\:}$and$\:\stackrel{\sim }{F}$were computed from the first *r* columns from the respective matrices obtained from singular value decomposition of *XD*^*− T*^, *D* was the Cholesky decomposition of *I + αB*, and *I* was the identity matrix.

4 $$\:{H}^{\mathrm{*}}=\stackrel{\sim }{E}$$

5 $$\:{W}^{\mathrm{*}}=\stackrel{\sim }{\varSigma\:}\stackrel{\sim }{{F}^{T}}{D}^{-1}$$

The resulting matrix W* used to determine typing regularity was obtained after setting *r* to 1 and *α* to 100. Varying the values of these variables did not drastically change our results. The signs in the resulting matrix W* were inverted if all values were less than 0 to obtain a positive matrix.

#### Sleep Estimation

The matrix W* was used to estimate the hours of each day that participants were asleep. First, W*, consisting of only values greater than or equal to zero, was binarized such than values greater than 0 were set to 1. Values of 0 were representative of no typing activity, while values of 1 indicated typical typing activity. Then, using a flood-fill algorithm from Python’s scikit-image package (version 0.19.2) [62] on the binarized matrix, the blocks of time that each individual typically did not type were identified. This approach identified the main consistent block of time that participants were usually asleep (i.e. hours surrounding 02:00–04:00). However, the algorithm treated the matrix as a two dimensional image with each row corresponding to the hours 00:00–23:00 of each day rather than one continuous block time segmented by date. As a result, any periods of sleep commenced before midnight were not identified using the flood-fill algorithm. To correct for these hours, consecutive hours from the previous day iterating backwards from midnight with values from the binarized matrix of 0 were also given a sleep label. Lastly, a sliding window of 8 h with a 2 h gap iterated through the labeled matrix, reshaped to size *1* × *observations* to allow for the continuation of time across days, to remove small gaps in sleep or wake labels for each day. The final matrix consisted of one continuous block of “sleep” and one continuous block of “wake” per relative 24 h period.

### Prediction of Processing Speed and Executive Function

#### Data Processing

Due to the naturalistic nature of the collected data, the quality and quantity of contributed data varied between participants. The first six (or less) dTMT-Bs completed per participant were initially included for analysis. Then, dTMT-Bs above the 95th percentile in either the number of errors made (≥ 6 errors) or the total time to completion (≥ 30.91 s) were also removed with the rationale that extremely long completion times or unusually high number of errors were likely the result of external or other factors unrelated to cognitive abilities, such as significant outside distractions or misunderstanding of task instructions. The remaining dTMT-Bs were matched to corresponding typing and accelerometer data by timestamp. For each dTMT-B, all corresponding participant keystrokes and accelerometer readings with timestamps within a six hour range around the dTMT-B timestamp were included in the analysis if at least 20 keystrokes were identified in this range. Following all filtering, there were a total of 397 dTMT-Bs from 94 participants used in the analysis.

#### Adjusted Trail-Making Test part B Time

The TMTs are known to be strongly affected by practice effects [53]. To control for this factor in our analysis, the task completion times were adjusted as follows. Prior to any filtering of tasks, the dTMT-Bs for each participant were numbered chronologically to designate the number of tasks that had been taken up until that task (i.e. 1st dTMT-B taken, 2nd dTMT-B taken, etc.). Then, following exclusion of tasks due to unusually poor performance (i.e. above 95th percentiles in errors made or time to completion) or insufficient corresponding typing data, dTMT-Bs across all participants were grouped by their task numbers. For each group, the dTMT-B times were z-scored such the mean across all groupings was 0 with a standard deviation of 1. This adjustment removed the differences in the task times solely due to the order at which they were taken by each participant. The models constructed in this study used this adjusted dTMT-B time as the measure of dTMT-B performance.

#### Feature Development

Features in this analysis comprised of typing and phone orientation related features thought to capture information pertaining to executive functioning and processing speed as well as the features thought to be associated with depressive symptoms as described in Table 1 [25, 26, 28]. Of note, typing speed was defined as the median time between consecutive keystrokes (denoted as interkey delay, IKD) in seconds between consecutive alphanumeric-alphanumeric transitions during two handed typing sessions. Since longer gaps between consecutive keystrokes (larger IKDs) are interpreted as slower typing speed, larger values of this feature indicate slower typing speeds. Additionally, orientation features were calculated based on a method previously developed which identifies personalized phone orientations from raw accelerometer data collected during typing sessions [63]. For all features, the typing metadata included for each calculation consisted of keystrokes and accelerometer readings within the six hours surrounding each dTMT-B with the exception of the feature calculation of the fraction of nighttime non-upright typing sessions. Computation of this feature incorporated typing and accelerometer metadata, filtered to only include data timestamped between the hours of 00:00–05:59, up to one week prior to each dTMT-B. All included features were both grand mean (gm) and subject centered (sc) to determine the effects of participants’ average feature value as well as fluctuations from personal average on dTMT-B performance.

**Table 1:** Data description of features derived from typing and accelerometer metadata

	Feature	Description
Accelerometer Features	nClusters	Number of individualized phone orientations used to type
	normalized_n_cluster_transitions	Sum of number of phone orientation changes normalized by number of typing sessions
	avg_n_cluster_transitions_perSession	Average number of times phone orientation changed during each typing session
	medianX	Median of absolute value of x-axis accelerometer reading
	fraction_nighttime_nonUpright_sessions	Fraction of typing sessions completed between the hours of 12:00–17:59 while typing in a non-upright position (X > 0.5 or X < −0.5 and Z ≥ 0.1)
	fraction_upright	Fraction of typing sessions completed while typing in an upright position (−0.2 ≤ X ≤ 0.2 and Z ≤ 0.1)
Typing Features	nSessions	Number of typing sessions
	avgNkeypresses	Average number of keypresses per typing session
	backspaceRate	Fraction of backspaces per total keypresses
	autocorrectRate	Fraction of autocorrect events per total keypresses
	typingSpeed	Median time between consecutive keystrokes (interkey delay) for alphanumeric-alphanumeric keystroke transitions for two-handed typing sessions
	varAAIKD	Variance in time between consecutive keystrokes (interkey delay) for alphanumeric-alphanumeric keystroke transitions
	medianPressDuration	Median time keys were pressed

#### Typing Regularity Features

To determine whether features pertaining to typing regularity and estimated sleep could improve model predictions, typing regularity features were derived from the matrix W* and associated predicted sleep labels, detailed in Table 2. These features were calculated using typing metadata collected up to one week prior to each dTMT-B.

**Table 2:** Typing regularity and estimated sleep feature descriptions

Feature	Description
varCircVar	Variance in the circular variance of the rows in matrix W* corresponding to variance in daily typing activity patterns
medAmt_noActivity/varAmt_noActivity	Median/variance in amount of estimated sleep quantity
medianCosineSimilarity_1diff/medianCosineSimilarity_4diff	Median cosine similarity between rows in matrix W* with 1 or 4 row gaps corresponding to comparison of typing activity patterns between days 1 or 4 apart

#### Prediction of Digital Trail-Making Test part B Performance

The dataset was divided into training and test sets using an 80/20 split by participant, which guaranteed that no participant had data in both the training and test sets. The test set was used at the end of model construction solely to evaluate the performance of the final model using data from previously unseen participants. Although the number of tasks contributed per participant had some variation, there was also an 80/20 split in the number of tasks within each set. To identify the features most likely to contribute to model performance, feature selection was performed using the mutual information (MI) filter-based method on the training set only [64, 65]. The top 10 of 36 features were selected for use in modeling by support vector regression (SVR), k-nearest neighbors (KNN), and random forest (RF). Models using SVR, KNN, and RF were constructed to predict adjusted dTMT-B time using the selected features. Least absolute shrinkage and selection operator (Lasso) regression modeling was also performed, using the intrinsic feature weighting and elimination to determine feature selection. All modeling used group 3-fold cross validation and Grid Search functionality (GridSearchCV function from the Scikit-Learn model_selection package, version 1.0.2) [67] for hyperparameter tuning (parameters shown in Supplemental Table 1) to improve model performance and prevent overfitting. All features were scaled prior to model construction for Lasso, SVR, and KNN methods. From the best performing model, determined by the lowest root mean squared error (RMSE) and mean absolute error (MAE) from the test set, the importance and directionality of features on model performance was obtained by calculating the SHapley Additive exPlanations (SHAP) values for each prediction using the SHAP package (version 0.39.0) [68, 69]. A baseline model constructed using the mean prediction of the training instances was used to compare to the performance of the best performing model.

### Effect of Mood on Model Performance

To determine if the passively-derived features implemented in this study were able to account for variance in the data relating to depressive symptoms, the best performing model was modified to include each participants’ average PHQ score, and the hyperparameters were re-tuned using the same gridsearch parameters to reduce the risk of overfitting. The model performance when including average PHQ score as a predictor was compared to the random forest model without this feature. To assess the importance of the average PHQ score in comparison to the other selected predictors, the SHAP values were calculated and plotted.

All processing was conducted in Python, version 3.7.4 [70] using the Pandas (version 1.3.5) [71, 72] and NumPy (version 1.17.2) [73] packages. Graph regularized SVD and estimated sleep labeling was computed with the SciPy package (version 1.1.0) [74]. Matrix visualization was done with the Matplotlib (version 3.5.3) [75] and Seaborn (version 0.9.0) [66] packages. Modeling was conducted using the Scikit-Learn package (version 1.0.2) [67].

## Results

Table 3 outlines the demographic and clinical information and data contributions of all participants. Due to increased requirements for amount of typing metadata to calculate the typing regularity and estimated sleep features, the models containing typing regularity and estimated sleep features consisted a subset of 62 participants with a total of 178 dTMT-Bs. The demographic and clinical information was similar between all participants and the subset of participants. All participants except one reported at least 12 years of education. Of the total participants included in each analysis, approximately 80% had been diagnosed with a mood disorder, though not all were actively experiencing symptoms during the study period. The average PHQ score across all participants was 6.09, suggesting an overall very mildly depressed sample. Closer examination of the individual PHQ item responses was done to determine how participants reported their overall sleep quality during the study. Of the 62 participants, the average sleep item score was 1.02 out of 3, meaning that participants had on average sleep difficulties for at least several days out of their reported retrospective period. Estimated sleep quantities for this subset, determined through typing activity, ranged from 1 to 11 h with a median of 6 h and interquartile range of 2 h.

**Table 3:** Participant demographic and clinical information for dTMT-B modeling

	Without Typing Regularity Features	With Typing Regularity Features
Number of Participants	93	62
Diagnosis (Mood Disorder (%))	73 (78%)	49 (79%)
Age	31.73 (6.14)	30.89 (5.93)
Gender (Female (%))	63 (67%)	44 (71%)
Education (≥ 12 years (%))	93 (99%)	62 (100%)
Average PHQ Score	6.09 (4.82)	6.56 (5.12)
Average PHQ Item 3 Score	0.93 (0.86)	1.02 (0.89)
Number Typing Sessions	26.53 (18.61)	32.46 (21.24)
Number dTMT-Bs	4.22 (1.62)	2.87 (1.35)

Values are reported as means and standard deviations unless otherwise denoted

*dTMT*-*B* digital trail-making test part B, *PHQ* Patient Health Questionnaire

### Estimated Sleep from Typing Activity

A method to capture diurnal patterns and sleep estimations from smartphone typing activity was developed using graph regularized SVD. Features engineered to capture information relating to typing regularity as a proxy for diurnal pattern were computed using the matrix W* and those designed to capture estimated sleep quantity and variability used the matrix containing predicted sleep and wake labels. Descriptions of these features are listed in Table 2.

### Feature Selection

To reduce the feature count and identify those most likely to contribute to model predictions, feature elimination was conducted for all models. The inherent feature elimination process applied during Lasso modeling did not weight any of the features supplied. For the SVR, KNN, and RF models, feature selection using MI identified the 10 features most likely to improve model performance while restricting the number of independent variables in the model, shown in Table 4 with summary statistics of these features shown in Table 5. The features selected related to phone orientation and its variability, typing speed, median press duration, and backspace rate, incorporating both grand mean and subject centered predictors.

**Table 4:** Mutual information (MI) values ascribed to the predictors before and after addition of typing regularity and estimated sleep features

Feature	MI Before Addition of Typing Regularity Features	MI After Addition of Typing Regularity Features
nClusters_sc	**0.092**	0.032
nClusters_gm	0.015	0.000
normalized_n_cluster_transitions_sc	0.003	0.000
normalized_n_cluster_transitions_gm	**0.082**	**0.161**
medianX_sc	0.010	0.000
medianX_gm	**0.082**	0.025
fraction_nighttime_nonUpright_sessions_sc	**0.064**	**0.083**
fraction_nighttime_nonUpright_sessions_gm	**0.119**	**0.088**
fraction_upright_sc	**0.065**	0.000
fraction_upright_gm	0.006	0.000
nSessions_sc	0.000	0.000
nSessions_gm	0.037	0.000
typingSpeed_sc	**0.100**	0.000
typingSpeed_gm	**0.048**	**0.102**
backspaceRate_sc	0.034	0.000
backspaceRate_gm	**0.063**	0.025
autocorrectRate_sc	0.000	0.000
autocorrectRate_gm	0.026	0.000
varAAIKD_sc	0.000	0.000
varAAIKD_gm	0.020	0.000
medianPressDuration_sc	0.045	0.000
medianPressDuration_gm	**0.080**	**0.124**
varCircVar_sc		**0.044**
varCircVar_gm		0.000
medAmt_noActivity_sc		**0.077**
medAmt_noActivity_gm		**0.059**
varAmt_noActivity_sc		0.000
varAmt_noActivity_gm		0.000
medianCosineSimilarity_1diff_sc		0.000
medianCosineSimilarity_1diff_gm		**0.063**
medianCosineSimilarity_4diff_sc		0.019
medianCosineSimilarity_4diff_gm		**0.192**

Selected features from each analysis in bold. All features are listed as both grand mean (*_gm) and subject centered (*_sc)

**Table 5:** Summary statistics of chosen predictors and target variable for all participants (*n*=93)

	Feature	Mean (SD)	Median	Min	Max
Target Variable	adj_TMT_time	0 (1.00)	−0.14	−2.15	3.67
Typing and Accelerometer Features	nClusters_sc	0 (1.06)	0	−4.2	4.67
	normalized_n_cluster_transitions_gm	0.17 (0.088)	0.17	0	0.5
	medianX_gm	0.089 (0.084)	0.065	0	0.48
	fraction_nighttime_nonUpright_sessions_sc	0 (0.33)	0	−0.83	0.83
	fraction_nighttime_nonUpright_sessions_gm	0.51 (0.31)	0.50	0	1
	fraction_upright_sc	0 (0.16)	0.013	−0.77	0.46
	typingSpeed_sc	0 (0.023)	−0.002	−0.068	0.12
	typingSpeed_gm	0.21 (0.055)	0.19	0.13	0.50
	backspaceRate_gm	0.12 (0.039)	0.12	0.036	0.24
	medianPressDuration_gm	0.083 (0.014)	0.083	0.055	0.12
Typing Regularity Features	varCircVar_sc	0 (0.00001)	0	−0.00011	0.00007
	medAmt_noActivity_sc	0 (0.88)	0	−4.5	3.5
	medAmt_noActivity_gm	5.99 (1.58)	6.5	1.25	11
	medianCosineSimilarity_1diff_gm	0.68 (0.11)	0.68	0.34	0.89
	medianCosineSimilarity_4diff_gm	0.65 (0.12)	0.65	0.29	0.93

Summary statistics for typing regularity features calculated from a subset of the participants with sufficient typing activity data (n=62)

* SD* standard deviation

Comparison of the MI between the shared features in the two models with and without typing regularity features revealed a general decrease in MI ascribed to the typing and orientational features following addition of typing regularity and estimated sleep parameters (Table 4). Half of the selected features sourced from those relating to typing regularity and estimated sleep.

### Model Results

#### Before Addition of Typing Regularity Features

To further optimize the models, hyperparameter tuning was conducted for all models tested. Aside from the following listed parameters, models were fit using the respective default parameters as defined by the Scikit-Learn functions (version 1.0.2) [67]. The Lasso model was tuned with an alpha of 0.5. SVR set the regularization parameter to 0.5 and the kernel to poly. KNN used the KDTree algorithm to compute the 30 nearest neighbors. Lastly, the RF model set the criterion to absolute error, the maximum depth of the trees to 4, the minimum sample to be a leaf to 5, the minimum sample to split a node to 3, and created 15 trees.

**Fig. 4:**
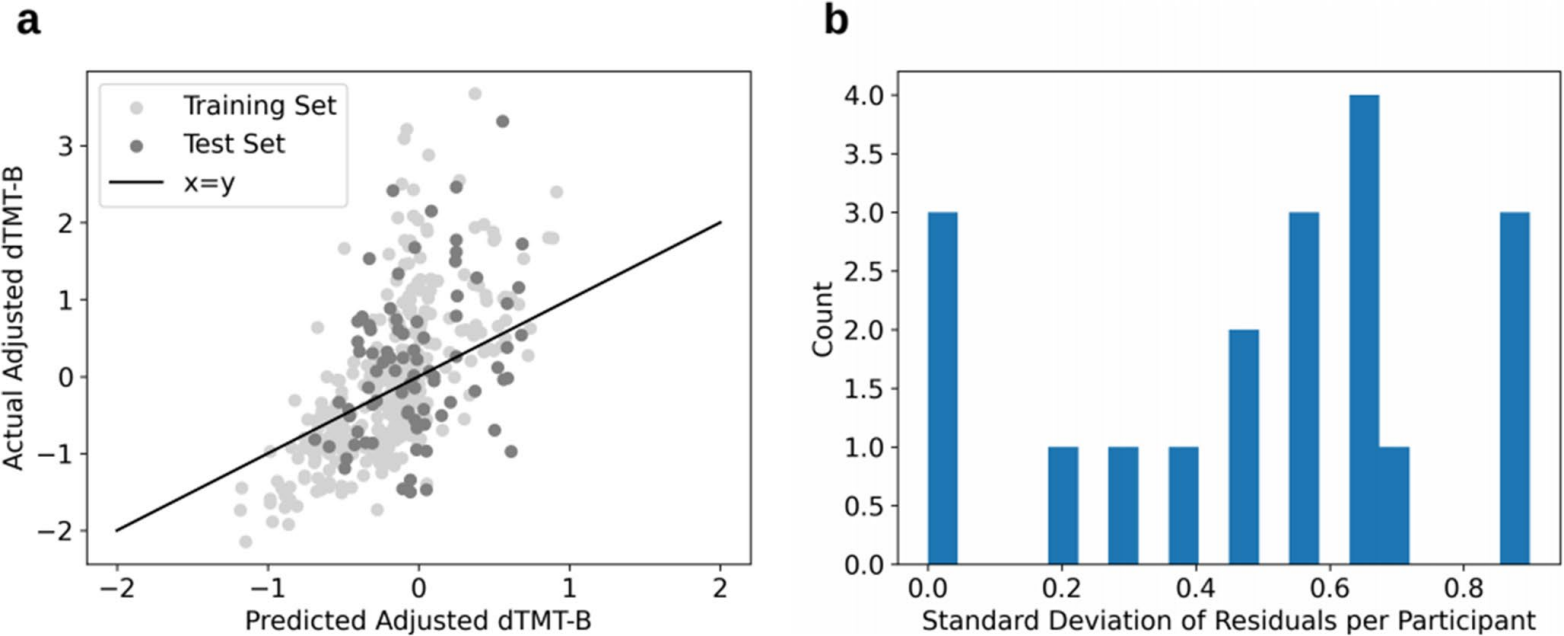
Plots of **a** predicted vs. actual adjusted dTMT-B scores for the training (light gray) and test (gray) sets, and **b** the spread of the standard deviations of the residuals per participant from participant data in the test set before the addition of typing regularity features

The performances of the four models evaluated using the test set are displayed in Table 6. Since no features were weighted for the predictions of adjusted dTMT-B time, the Lasso model predicted all tasks to have the same time, and thus was not a good fit of the data. Of the remaining methods, the RF model performed the best with the lowest RMSE of 0.938, MAE of 0.724, explained variance score of 0.105, and R^2^ of 0.064. Since the predicted values were z-scored, this error is reported in standard deviations from the mean as opposed to raw time in seconds. A plot of the individual model predictions for both the training test sets is shown in Fig. 4a, and the within-subject variability is shown in Fig. 4b through the standard deviation of the residuals per participant in the test set only. A RF baseline model constructed to compare to the performance of the RF model showed consistent reduction in error and improved explained variance and R^2^ scores with an approximate 4.4% reduction in RMSE (Table 6).

**Table 6:** Model results from prediction of adjusted dTMT-B performance before and after addition of typing regularity features

Model	Before Addition				After Addition			
	RMSE	MAE	Explained Variance Score	*R* ^2^	RMSE	MAE	Explained Variance Score	*R* ^2^
Lasso	0.994	0.761	0.0	−0.052	–	–	–	–
SVR	1.047	0.812	0.009	−0.166	0.906	0.782	−0.412	−0.457
KNN	0.968	0.732	0.068	0.002	0.817	0.717	−0.025	−0.184
RF	0.938	0.724	0.105	0.064	0.769	0.644	0.016	−0.050
Baseline RF (mean prediction)	0.994	0.761	0.0	−0.051	0.804	0.669	0.0	−0.148

*Lasso* least absolute shrinkage and selection operator, *SVR* support vector regression, *KNN* k-nearest neighbors, *RF* random forest, *RMSE* root mean squared error, *MAE* mean absolute error, *dTMT*-*B* digital trail-making test

#### After Addition of Typing Regularity Features

**Fig. 5:**
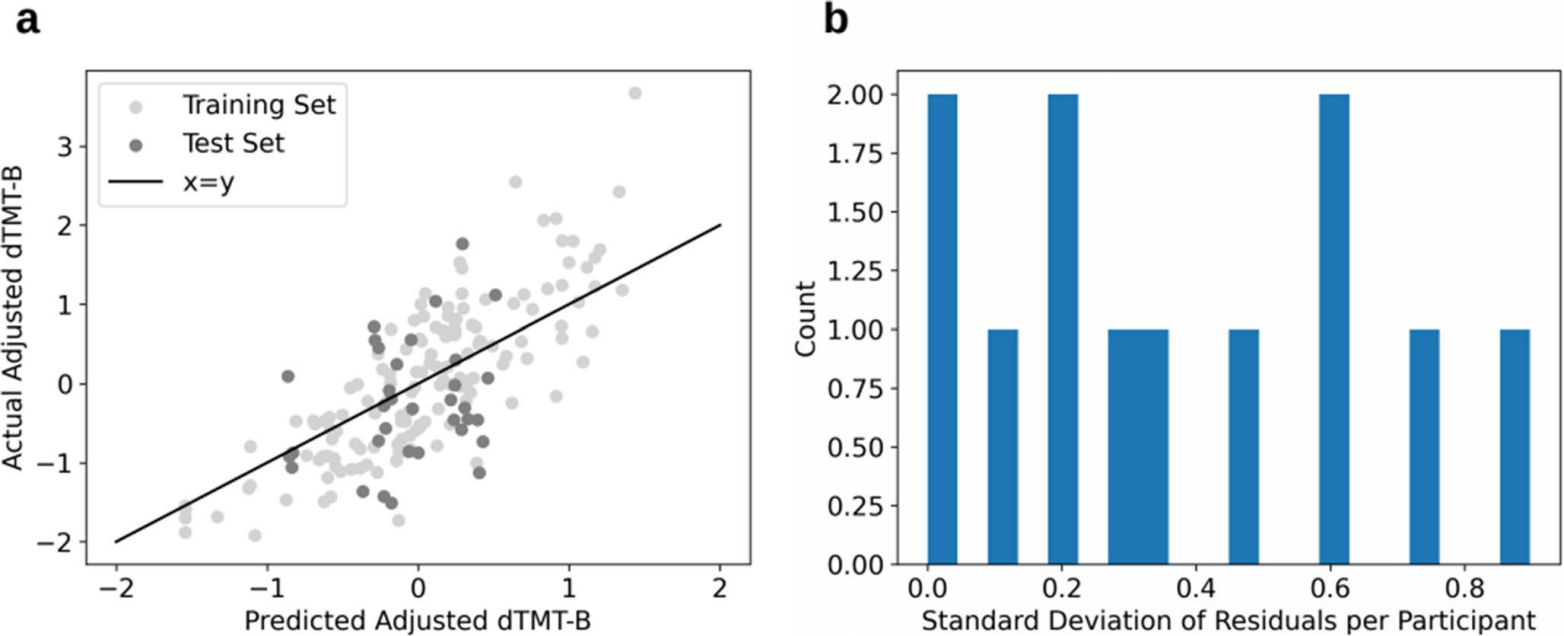
Plots of **a** predicted vs. actual adjusted dTMT-B scores for the training (light gray) and test (gray) sets, and **b** the spread of the standard deviations of the residuals per participant from participant data in the test set after the addition of typing regularity features

Models tested with the new set of features included SVR, KNN, and RF. SVR utilized the default parameters. The Lasso model was not included due to poor model fit described above. The KNN model was tuned using the BallTree algorithm and 30 nearest neighbors. RF hyperparameter tuning used the default criterion, a maximum depth of 5, minimum samples to be a leaf of 4, minimum samples to split a node of 3, and 30 generated trees. Model results are shown in Table 6. Of the other models, the random forest model had the lowest RMSE and MAE but once again with low explained variance and R^2^ scores. Fig. 5a shows the individual predictions for the training and test sets, and Fig. 5b illustrates the within-person variability through the standard deviations of the residuals per participant in the test set only. The addition of the typing regularity features resulted in an improved model performance with a reduction in RMSE from 0.938 to 0.769 and in MAE from 0.724 to 0.644, suggesting that the combination of these features was able to capture more information regarding processing speed and executive functioning than typing and orientational features alone. Comparison of this RF model to its baseline model performance also showed a consistent decrease in error and increase in explained variance and R^2^ scores with an approximate 5.6% reduction in RMSE (Table 6).

### Feature Importance and Directionality

#### Before Addition of Typing Regularity Features

**Fig. 6:**
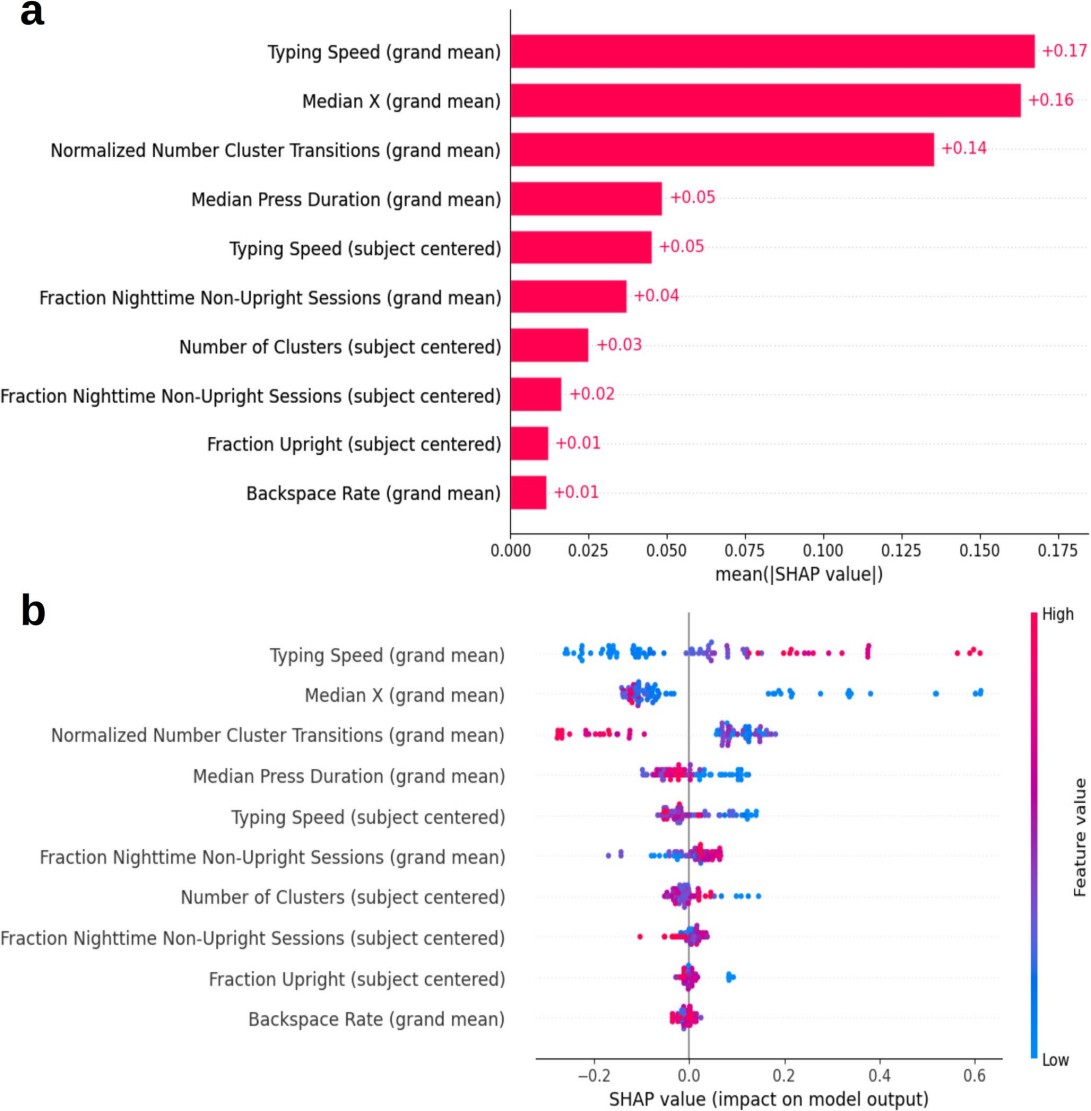
Plots of SHAP values showing **a** the mean absolute SHAP value per feature as a measure of feature importance in model predictions of adjusted dTMT-B performance and **b** individual SHAP values colored by corresponding feature values to indicate directionality of features on model predictions of adjusted dTMT-B times

To better understand how each feature contributed towards the model predictions, SHAP values were obtained from the RF models. These values, developed from the principles of game theory, describe how much each feature changed the individual model predictions from their expected outputs. Every model prediction is given a set of SHAP values, one from each feature. The sum of these values equates to the deviation from the base model prediction to the observed prediction. For a regression model, the sum of these values equal the deviation from the base model output (in this case, the mean adjusted dTMT-B time) to the observed model output. By calculating the mean absolute SHAP value for each feature, a feature ranking could be obtained, depicted in Fig. 6a. There were three features that dominated the model predictions, which will be discussed in further detail. Typing speed (grand mean centered) and the accelerometer median x-axis reading (grand mean centered), a measure of how vertical or horizontal the phone was during typing sessions, were found to impact model prediction the most with mean absolute SHAP values of 0.17 and 0.16, respectively. The normalized number of cluster transitions (grand mean centered), which measured how often the phone changed phone orientation during consecutive typing sessions, ranked closely behind with a mean absolute SHAP value of 0.14. The other features all contributed some to the model as well, though to a lesser degree.

The trends between the feature values and resulting model prediction allowed for interpretation of the directionality of the feature’s impact on the model, with lower SHAP values corresponding to faster dTMT-B times and better cognitive performance, shown in Fig. 6b. The trends were generally linear, allowing for easier interpretation. First, there was a distinct linear trend observed for average typing speed, in which those who typed more quickly (illustrated by larger values due to our calculation of typing speed) also had faster corresponding adjusted dTMT-B times. Interestingly, fluctuations in typing speed within each participant (subject centered typing speed) showed the opposite trend, in which those who typed more quickly compared to their average speed (had a smaller feature value) completed the corresponding dTMT-Bs more slowly than their average completion time. Second, there was a general relationship between participants who on average had a larger accelerometer median x-axis reading, corresponding to more horizontal phone orientations on average compared to other participants, and faster adjusted dTMT-B performances. This pattern however, did not hold true for all model predictions. Lastly, participants who on average more frequently transitioned between cluster centers (i.e. unique phone orientations for each individual) and typed in an upright position at night more often generally performed better on the dTMT-B.

#### After Addition of Typing Regularity Features

**Fig. 7:**
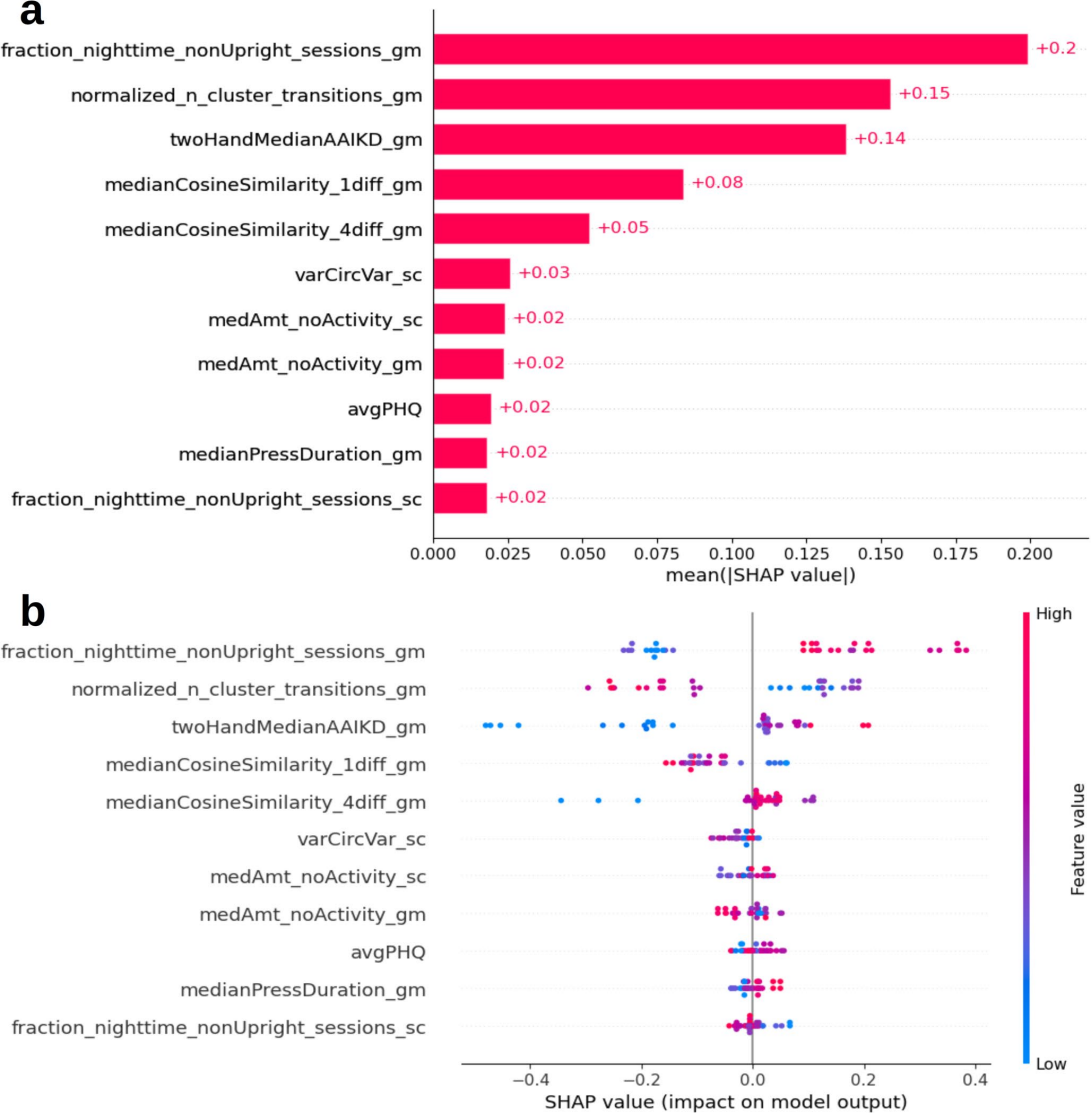
Plots of SHAP values depicting **a** the mean absolute SHAP value per feature as a measure of the influence of each feature on model predictions and **b** the individual SHAP values colored by feature value to illustrate trends between feature values and model outcomes

The importance and directionality of the features on model prediction is shown in Fig. 7. The fraction of nighttime non-upright typing, normalized number of cluster transitions, and typing speed (all grand mean centered) contributed most to model predictions with mean absolute SHAP values of 0.2, 0.15, and 0.14, respectively (Fig. 7a). Typing regularity features relating to the similarity in typing patterns between consecutive and every 4 days (median cosine similarity with 1 and 4 day gaps) had mean absolute SHAP values of 0.09 and 0.06, respectively, with the other features contributing to model predictions less. Directionality of the features used in the model described above remained the same in this model with more phone orientation transitions, less non-upright nighttime sessions, and faster typing speeds being associated with better dTMT-B performance (Fig. 7b). Directionality of moderately contributing typing regularity features was as follows: higher median cosine similarity between typing patterns across consecutive days related to better dTMT-B performance, while higher median cosine similarity between typing patterns across every 4 days was associated with worse dTMT-B performance when compared between participants. This suggested that similar patterns between consecutive days but more variability between days farther apart related to better processing speed and executive functioning.

#### Effect of Mood on Model Performance

**Fig. 8:**
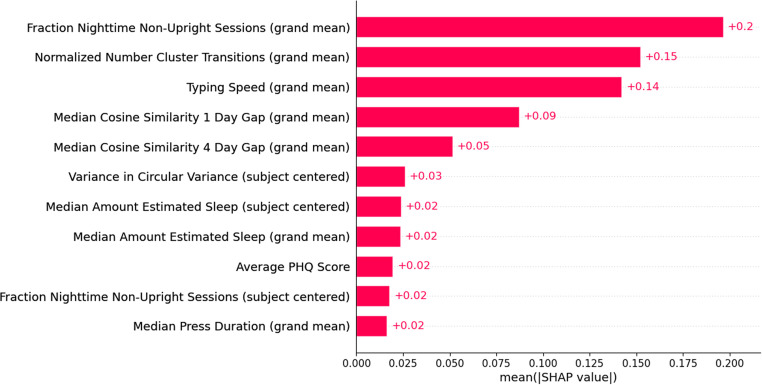
Plot of mean absolute SHAP values for each feature obtained from the random forest model predictions incorporating average PHQ score

The ability for smartphone typing-based behaviors to capture mood as related to its effect on dTMT-B performance was assessed, since a previous analysis conducted showed that depression severity, in which a direct measurement was excluded from the models discussed here, was associated to dTMT-B performance [31]. This assessment was conducted by the addition of average PHQ score to the best performing model described above to observe for any model improvement. Using the same sample and an 80/20 training/test split by participant and number of tasks, average PHQ score for each participant was added to the features used in the second RF model, and a new RF model was optimized using the same parameters. The resulting model RMSE and MAE were 0.769 and 0.654, which was approximately equal to the error in the second model which excluded the average PHQ score predictor. Calculated SHAP values indicated the model only minimally relied on average PHQ score to predict the adjusted dTMT-B times (Fig. 8), suggesting that the typing-related features used in the model accounted for the variance in the data that would otherwise have been attributed to mood.

A further investigation to assess for any model dependence on measures of depressed mood examined the addition of the average PHQ scores from the first two questions alone, which specifically addressed feelings of anhedonia and depressed mood. RF model performance was very similar to that of the model which included average PHQ score with all items in both error and feature importance (data not shown).

## Discussion

The goal of this study consisted of two parts: first to develop a method to estimate diurnal patterns and sleep exclusively from typing activity and engineer features capturing this information, and second, to evaluate the feasibility of unobtrusively monitoring processing speed and executive functioning using only information gathered through smartphone typing behaviors and assess the utility of the novel diurnal features to improve model predictions. A few studies have also explored phone activity to estimate sleep duration with all showing approximate agreement between various self-reported sleep measures and phone activity [47–49]. To the best of our knowledge however, this is the first to incorporate the impact of surrounding days on daily estimates in a sample outside of a university population. Our objective to approximate diurnal patterns and sleep using only typing activity was to balance the advantages and disadvantages between current subjective and objective measures. In addition to not directly measuring physical movement, typing activity was sporadic and so would be less accurate than any form of continuous measurement, but would not require active engagement or for participants to remember to continuously wear an additional device. Typing regularity and the extensive absence of typing activity in this study served to capture behaviors influenced by diurnal patterns and sleep instead of the patterns and quantities themselves. Nevertheless, using the method outlined in Fig. 2 and conceptually visualized in Fig. 3, the majority of estimated sleep quantities ranged between 5 and 7 h. These estimates align with typical hours of sleep duration in adults when considering that many reported at least some disturbances in sleep [76, 77].

The utility of our diurnal pattern and sleep approximations were evaluated through the comparison of models predicting processing speed and executive function, as measured by a digital TMT-B (Fig. 1), in a sample of individuals with and without depressive symptoms (Table 3). Practice has been well-known to be a source of confound to the interpretation of TMT-B performance due to the probable improvement following repeated task completions [53]. As this study included repeated administrations of the task, practice effects were expected. To control for this source of improvement, dTMT-B times were standardized to allow for the evaluation of differences due to factors pertaining to the cognitive abilities of the participants. This normalization technique has been used in previous studies to control for other confounds, such as age and education level [44, 78], and was chosen over the alternative of including a practice variable in the model to refrain from constructing a model containing task-derived measures to predict task performance.

To predict the performance of executive functioning and processing speed as measured by the dTMT-B, feature selection was first performed to consolidate the predictors in the models from the combined list in Tables 1 and 2 to those thought to be most relevant. For the first model excluding typing regularity and estimated sleep features, the selected variables included typing speed, both average and within subject fluctuations, which previously had been found to be significantly associated with dTMT-B performance [31], as well as an even amount of features relating to behaviors surrounding the number and frequency of phone orientations chosen while typing and those pertaining to typing-specific dynamics (Table 4 and 5). Interestingly, grand mean centered variables, which allow for comparison between participants’ average parameters to one another, were chosen more often than subject centered features, which examine within participant fluctuations on performance. The reliance on between subject differences for model predictions may suggest that one of the biggest predictors was the individual themself. This notion is supported by studies emphasizing personalized models for enhanced predictions of mood and cognition [20, 34, 79, 80].

The features derived from typing regularity and estimated sleep extracted relationships between typing patterns across neighboring days as well as typical gaps in typing activity over time (Table 2). Upon inclusion of these features in the list of possible predictors, many were chosen by the feature selection method over previously selected typing and orientational features, shown in Table 4. The shift to these typing regularity features supports the notion that sleep and circadian rhythm are related to aspects of cognitive functioning [81] and can be passively captured from smartphone typing activity.

Prior to the addition of predictors relating to diurnal patterns and sleep, the RF model had the lowest RMSE and MAE of 0.938 and 0.724, respectively, of the four modeling methods tested, shown in Table 6 and visualized in Fig. 4. Since the predicted values had been z-scored, these errors corresponded to standard deviation units as opposed to raw values. For these models, we observed low explained variance and R^2^ scores. These values may be in part due to a combination of factors stemming from the model being tested on data from previously unseen participants and the z-scored target variable. We z-scored dTMT-B completion times within each task number grouping (e.g., first administration, second administration, etc.) to control for practice effects. This adjustment primarily reduces within-subject variance attributable to repeated exposure, thereby ensuring that remaining within-subject variability reflects other factors (e.g., fatigue, diurnal fluctuations, mood state, environmental distractions). Between-subject differences are preserved within each task number, but they are re-scaled to a common standard deviation. Because the standardization is performed separately for each task number, any between-subject variance arising from consistent individual differences in practice-related gains is also attenuated. Thus, the procedure removes the confounding influence of practice while retaining scaled between-subject differences in performance for each administration. Although removing the confound of practice improved the interpretability of our model, the resulting low variance in the target variable can affect explained variance and R² scores and may lead to misleading scores when evaluating model performance [82, 83]. Our findings suggested that through analyzing solely information collected from an individual’s naturalistic typing behaviors, aspects about their cognitive performance, otherwise measured by the dTMT-B, could be estimated within less than one standard deviation of the mean on the dTMT-B. Furthermore, these predictions were impartial to practice effects, unlike the conventional TMT-B [53]. Although this concept of analyzing smartphone typing as an assessment of cognitive function is fairly new in the literature, a few other studies conducted in patient populations such as Alzheimer’s disease/mild cognitive impairment and multiple sclerosis have also concluded that harnessing smartphone typing dynamics may have the potential to elucidate the impairments in underlying cognitive processes [30, 32, 58, 84, 85].

As hypothesized, we obtained better RF model performance following the addition of typing regularity features (Table 6 and Fig. 5), suggesting that our method of extracting diurnal patterns and estimated sleep from typing activity related to participants’ processing speed and executive function. For both of our RF models, we observed an approximate 5% lower RMSE compared to their corresponding baseline models. However, a paired permutation test on the absolute residuals in the test fold did not yield statistically significant differences (*p* > 0.05). This small, non-significant reduction in error was observed even after removing variance through z-scoring the target variable to control for practice effects and testing the model on data from previously unseen participants. While not statistically significant, the improvement was consistent across all error metrics, which we interpret as preliminary support for the mechanistic role of typing-related and diurnal activity features in cognitive performance. We note that larger samples may be needed to detect statistically reliable differences in predictive accuracy.

Evaluation of the calculated SHAP values for each prediction in the model allowed for determination of feature importance and directionality. Before the addition of typing regularity and estimated sleep features to the model, three features contributed the most to model predictions: typing speed (grand mean centered); accelerometer’s median X-axis reading (grand mean centered), which measured how horizontal the phone was while typing; and normalized number of cluster transitions (grand mean centered), which measured how often the phone changed orientation across consecutive typing sessions (Fig. 6a). After the addition of the typing regularity features, those relating to typing speed and phone orientation and transitions remained the greatest contributors to predicting dTMT-B performance (Fig. 7a). In comparison, typing regularity features were observed to be of moderate importance for model predictions, which suggested that typing regularity (as a proxy for diurnal patterns) did not capture as much information relating to processing speed and executive function as other typing-related behaviors.

Trends in feature directionality revealed similarities between the two models, shown in Figs. 6b and 7b. First, examination of trends in typing speed, thought to relate most to the cognitive aspects of dTMT-B performance, suggested that participants who typed more quickly on average compared to the other participants were predicted to have better dTMT-B performance, which aligned with previous findings [31, 86]. The observed trend supports the notion that typing speed may be a naturalistic measure of processing speed [30]. However, without an isolated measure of processing speed, such as through the TMT part A (a digitally adapted version is not included on the BiAffect app), which consists solely of connecting numbers in ascending order, we are unable to confirm this hypothesis in our sample. Further work would be needed to unpack how our features relate to the specific aspects of cognition involved during the dTMT-B task.

A less defined trend for within subject fluctuations in typing speed in the first RF model revealed an opposite effect in which those who typed more slowly compared to themselves performed better than their average dTMT-B performance. One possible explanation for this juxtaposition could be related to a speed accuracy trade-off as has been seen in smartphone typing between typing speed and correction (autocorrect and backspace) rates [30]. If participants were typing more quickly than usual, they might also be tapping more quickly through the dTMT-B but making more errors along the way. The increase in errors would increase the task completion time, thus ultimately causing them to be slower than their average on the task. Without a decipherable measure of typing error in the model, however, we are unable to further investigate this possibility. This effect overall contributed less to the prediction of dTMT-B times, but more work is needed for proper interpretation of this finding and determination of its generalizability.

Furthermore, both models suggested that more transitions between varying phone orientations during consecutive typing sessions related to better dTMT-B performance, but varying trends in the top predictors relating to amount of non-upright typing sessions was observed between the two models, as seen in the comparison between the accelerometer’s median x-axis reading in Fig. 6b and the fraction nighttime non-upright sessions in Fig. 7b. However, nuances in the calculations, such as timing-related differences, between the specific features in the two analyses could attribute to the discrepancies in the interpretation of these results. Indeed, Ning et al. found that performance on the dTMT-B varied by time of day [86], so calculations of typing features based on varying timeframes may lead to confounding results. This is further supported by the fact that the predictor fraction nighttime non-upright sessions showed consistent directionality between the two models. Overall, these findings suggested that more flexibility in the chosen phone orientations while typing throughout the day was related to better processing speed and executive function, which might have related to levels of activity and the corresponding influence of depressive symptoms. Previous studies have found that increased sedentary behavior and limited physical activity is associated with greater depressive symptoms [87], which is known to impact cognitive performance [5].

The examination of the directionality of the typing regularity features in the second model suggested that similarity in typing patterns between consecutive days was associated with better cognitive performance, but similarity in typing patterns compared 4 days apart related to poorer performance. One rationale behind this finding might relate to the weekly schedules than many people have in which work schedules dominate time on weekdays and weekends allow for more variety in activities. Within this structure, typing patterns may consistently be more similar between consecutive days, while observations 4 days apart allows for more comparisons between weekdays and weekends. In line with this theory, Huber and Ghosh noted 7-day periodicity in smartphone usage in addition to the expected 24-hour cycle in their analysis of behavioral patterns across varying timeframes [88]. Disruption to this cycle could indicate the presence of a mood disorder and corresponding cognitive impairment. Indeed, those with major depressive disorder and bipolar disorder have been found to have increased absences from work and an overall loss of work performance [89], which suggests that their daily and weekly rhythms may fluctuate more than those without a mood disorder and thus have more cognitive dysfunction.

Finally, we have previously observed that depressive symptoms are significantly associated with dTMT-B performance [31]. Therefore, we verified that the selected features in our model also accounted for the effects of depressive symptoms on dTMT-B performance by comparison of the RF model performance following the addition of participants’ average PHQ score to the model. The addition of a measure of depression did not improve model predictions. Further examination of the feature importance on model predictions revealed that the average PHQ feature had limited contribution, shown in Fig. 8. These factors suggest that our derived smartphone typing behaviors capture information relating to the effect of depressive symptoms on dTMT-B performance and supports the use of a predictive model of processing speed and executive functioning entirely comprised of passively-derived features.

This analysis does comes with limitations. First, the dataset used in this analysis consisted solely of iPhone users. While the iPhone dominates the US market, this user base is not representative of the global population, which may limit the generalizability of our findings.

Second, as described before, the proxies for diurnal patterns and sleep obtained solely from typing activity requires caution in its interpretation due to the lack of knowledge about the activities the participants were engaged in when no typing activity is recorded. Typing regularity was used as a proxy for diurnal patterns; however, although the respective features related to executive functioning as hypothesized, the link between typing regularity and diurnal rhythm itself still needs to be established. Furthermore, estimated sleep labels were based on many assumptions. First, we assumed that participants did not take naps, and they were predicted to have typed on their smartphones right before they went to sleep as well as immediately after they woke up the next morning. Moreover, approximated sleep was based on hourly intervals and was not able to accommodate more precise sleep onset and wake times. Nonetheless, our approximations for the diurnal rhythms of the participants in this study produced coherent relationships with dTMT-B performance in line with the literature. However, future work should investigate the extent to which typing activity can approximate sleep and diurnal patterns in a diverse sample.

Additionally, contrary to traditional in-person assessments, the environmental variables in which the dTMT-B was collected could not be known. The remote administration of the dTMT-Bs, although convenient for the participant, meant that the environment in which they completed the tasks most likely varied between tasks and participants. This may result in better alignment between naturalistic smartphone typing and cognitive assessment in-the-wild and provide more ecological validity to the assessment, but further work would be needed to delineate these effects.

Moreover, the dTMT-B times in this study were adjusted to control for practice effects. The standardization was performed on groups with quantities ranging between 43 and 77 tasks, which might not have encompassed the true distribution of dTMT-B times for each sequential task. Furthermore, the standardization method assumed the same learning rate for all participants, which may not have been an accurate adjustment for each individual.

Lastly, TMTs generally comprise of two parts: part A and B. Our study consisted solely of part B, which meant that we were unable to separate processing speed from set-shifting (the cognitive process required by the task when switching between numbers and letters) in our analyses. However, one may expect that the ability of set-shifting is relevant in naturalistic typing (e.g., switching between QWERTY and special character layouts). Nevertheless, further work should be done to determine the clinical applicability of these findings to the clinical population.

## Conclusion

The present study supports the utility of the smartphone’s keyboard as a medium to passively measure processing speed and executive function without the need for demographic or clinical input. The derived metrics collected in-the-wild did not place any extra time demand on the participant, thus providing a possible unobtrusive modality to monitor changes in these aspects of cognition at a higher granularity. In addition, we present a novel method to extract information pertaining to diurnal patterns and sleep through an entirely unobtrusive data source and its relevance to predictions of processing speed and executive function. Relationships between typing regularity patterns and these aspects of cognitive functioning enhanced model predictions, which supports the feasibility of using smartphone typing dynamics to capture aspects of regularity for estimation of select domains of executive functioning in those with mood disorders.

## Supplementary Information

Below is the link to the electronic supplementary material.

Supplementary Material 1

## Data Availability

The data presented in this study are available upon request from the corresponding author. The data are not publicly available due to privacy concerns.
