# Supplementary material for: Keying Into Cognition: Temporal Smoothing of Smartphone Typing Behaviors for Passive Assessment of Processing Speed and Executive Function in Individuals With Mood Disorders
Source: Cognit Comput. 2026 Apr 1;18(1):33. doi: 10.1007/s12559-026-10549-y (PMC13043584; doi:10.1007/s12559-026-10549-y)
Supplement: Supplementary file 1 — Supplementary Material 1 [file 12559_2026_10549_MOESM1_ESM.pdf]

Supplemental Table I. Grid Search hyperparameters tested.

Model	Hyperparameter	Values Tested
Lasso	alpha	0.001, 0.01, 0.1, 0.2, 0.5, 0.7, 0.9, 1
SVR	kernel	‘linear’, ‘poly’, ‘rbf’
	C	0.05, 0.1, 0.5, 1, 2, 3, 5
	gamma	‘scale’, ‘auto’
KNN	n_neighbors	2, 3, 4, 5, 10, 20, 30
	algorithm	‘ball_tree’, ‘kd_tree’
RF	n_estimators	10, 15, 20, 30, 40, 50
	criterion	‘absolute_error’, ‘squared_error’
	max_depth	3, 4, 5
	min_samples_leaf	3, 4, 5
	min_samples_split	3, 4, 5

Abbreviations. Lasso = least absolute shrinkage and selection operator, SVR = support vector regression, KNN = k-nearest neighbors, RF = random forest.
